# Fireworks explosion boosted Harris Hawks optimization for numerical optimization: Case of classifying the severity of COVID-19

**DOI:** 10.3389/fninf.2022.1055241

**Published:** 2023-01-25

**Authors:** Mingjing Wang, Long Chen, Ali Asghar Heidari, Huiling Chen

**Affiliations:** ^1^School of Computer Science and Engineering, Southeast University, Nanjing, China; ^2^The Key Laboratory of Computer Network and Information Integration, Southeast University, Ministry of Education, Nanjing, China; ^3^School of Surveying and Geospatial Engineering, College of Engineering, University of Tehran, Tehran, Iran; ^4^College of Computer Science and Artificial Intelligence, Wenzhou University, Wenzhou, China

**Keywords:** Harris Hawks optimization, fireworks algorithm, numerical optimization, CEC2014 benchmark functions, COVID-19

## Abstract

Harris Hawks optimization (HHO) is a swarm optimization approach capable of handling a broad range of optimization problems. HHO, on the other hand, is commonly plagued by inadequate exploitation and a sluggish rate of convergence for certain numerical optimization. This study combines the fireworks algorithm's explosion search mechanism into HHO and proposes a framework for fireworks explosion-based HHo to address this issue (FWHHO). More specifically, the proposed FWHHO structure is comprised of two search phases: harris hawk search and fireworks explosion search. A search for fireworks explosion is done to identify locations where superior hawk solutions may be developed. On the CEC2014 benchmark functions, the FWHHO approach outperforms the most advanced algorithms currently available. Moreover, the new FWHHO framework is compared to four existing HHO and fireworks algorithms, and the experimental results suggest that FWHHO significantly outperforms existing HHO and fireworks algorithms. Finally, the proposed FWHHO is employed to evolve a kernel extreme learning machine for diagnosing COVID-19 utilizing biochemical indices. The statistical results suggest that the proposed FWHHO can discriminate and classify the severity of COVID-19, implying that it may be a computer-aided approach capable of providing adequate early warning for COVID-19 therapy and diagnosis.

## 1. Introduction

Numerical optimization is currently a hot topic for research in both science and technology. It is becoming increasingly difficult to deal with these issues with discontinuous, non-convex, and non-differentiable properties (Cui et al., 2017). In many cases, traditional gradient-based methods are ineffective because of their stringent use conditions and local convergence. As a result of these and other natural phenomena, many meta-heuristic algorithms (MHAs) have been developed in the last few decades by researchers around the world. Genetic algorithm (GA) (Mathew, 2012), differential evolution (DE) (Price, 2013), particle swarm optimization (PSO) (Poli et al., 2007), ant colony optimization (Paniri et al., 2020), and harris hawks optimization (HHO) (Heidari et al., 2019) are some of the most popular MHAs. For numerical optimization problems, these algorithms have proven to be extremely effective.

HHO (Heidari et al., 2019) is a meta-heuristic algorithm with population theory that mimics harris hawks' intelligent hunting behavior. HHO is proposed to assault the rabbit in four distinct ways: soft besiege, hard besiege, soft besiege with progressive rapid dives, and hard besiege with progressive rapid dives. Compared to other optimizers, HHO has a small number of parameters and a high capacity for exploration. Due to its simplicity, ease of implementation, and performance, HHO has garnered considerable attention and has been applied to a variety of real-world optimization problems, such as job-shop scheduling (Li C. et al., 2021), internet of vehicles application (Dehkordi et al., 2021), engineering optimization (Kamboj et al., 2020), and estimation of photovoltaic parameters (Chen et al., 2020b; Jiao et al., 2020). Although HHO has demonstrated competitive performance on a variety of problems when compared to other algorithms, such as PSO and GA, it still has some shortcomings in terms of exploitation (Alabool et al., 2021). However, an efficient search strategy should strike a good balance between global exploration and local exploitation, favoring exploration at first and turning to exploitation as the iteration counts increase. Thus, the development of a new search strategies to improve HHO's exploration–exploitation balance is crucial for increasing its performance on difficult numerical optimization tasks.

The fireworks algorithm (FWA) was developed by Tan and Zhu (2010) as a novel MHA technique. The fireworks explosion search is FWA's most productive operator, as it generates new individuals in the vicinity of several promising individuals that have been evenly distributed. The old will be replaced by new, more fitting individuals. FWA simulates a global search by bursting fireworks. The method possesses (1) an explosive search pattern and (2) a framework for the interaction of many (sub)populations. Since its conception, the FWA has drawn considerable research and has been widely applied to real-world optimization, most notably as a minimalist global optimizer (Li and Tan, 2018), multimodal function optimization (Li and Tan, 2017), and flowshop scheduling (He et al., 2019).

In this article, we propose a framework for fireworks explosion-based Harris Hawks optimization (FWHHO), which incorporates fireworks explosion search into the HHO algorithm. To be more specific, the FWHHO framework recommends two stages of search: one for hawks and another for fireworks explosions. After completing the four hawks' search phases soft besiege, hard besiege, soft besiege with progressive rapid dives, and hard besiege with progressive rapid dives, exploitation of prospective places is accomplished by the use of a fireworks explosion search. From populations, some individuals with a widespread are picked, and new individuals are generated in their vicinity. Experiments on CEC2014 benchmark functions reveal that using fireworks explosion search to optimize HHO algorithms can greatly enhance their performance. The FWHHO algorithm outperforms state-of-the-art meta-heuristic algorithms on the CEC2014 benchmark functions. Furthermore, the proposed FWHHO framework is applied to four existing HHO algorithms and fireworks algorithms, and the experimental results demonstrate that FWHHO shows the obvious property over existing HHO algorithms and fireworks algorithms. Finally, the proposed FWHHO is used to evolve a kernel extreme learning machine for the purpose of diagnosing COVID-19 using biochemical indexes. The main contributions of this study are as follows:

- The fireworks explosion-based Harris Hawks optimization is proposed in this article (FWHHO), and fireworks explosion operators are integrated into the original HHO.
- The FWHHO's performance is validated using CEC2014 benchmarks, and its capabilities clearly outperforms the original HHO.
- The FWHHO method considerably outperforms state-of-the-art techniques, existing HHO algorithms, and a variety of fireworks algorithms.
- The FWHHO can evolve a kernel extreme learning machine for the purpose of diagnosing COVID-19 using biochemical indexes successfully.

The following sections describe the structure of this article. Section 2 summarizes HHO algorithm research. Section 3 contains a detailed discussion of the FWHHO algorithms. Section 4 gives the details of the experimental designs. The results of the experiments are analyzed and discussed in Section 5. Section 6 includes the conclusion and future work.

## 2. Literature survey

Numerous improved HHO algorithms have been developed in recent years to improve performance. These algorithms can be classified into two categories: modification of HHO with new search equations and hybrid with other metaheuristic algorithms.

(1) Modification of HHO with new search operators: The search operators in HHO are used to direct the search and develop new solutions. Numerous new search operators have been developed to enhance the search capacity of the HHO. Song et al. (2021) proposed a new GCHHO with Gaussian mutation, and a dimension-decision strategy that was used in the cuckoo. The results show that GCHHO is very good at getting better results. A hybrid QRHHO algorithm was created by Fan et al. (2020). Fan et al. combined the exploration of HHO with the use of a quasi-reflection-based learning mechanism (QRBL). Gupta et al. (2020) suggested an opposition-based learning-based HHO (m-HHO), in which OBL improves the search efficiency of HHO and alleviates the problems of a standstill at suboptimal solutions and premature convergence. Zheng-Ming et al. (2019) developed a more efficient HHO algorithm based on the tent map. The results indicate that the tent map has the potential to enhance the HHO algorithm's capabilities. Qu et al. (2020) utilized information sharing for HHO and information from the cooperative foraging area and collaborators' location areas, so establishing that information exchange is shared. Ridha et al. (2020) introduced a boosted HHO (BHHO) algorithm with random exploratory steps and a strong mutation scheme, in which methods not only accelerate the convergence rate of the BHHO algorithm but also aid it in scanning additional parts of the search basins. Devarapalli and Bhattacharyya (2019) proposed a modified HHO algorithm with a squared decay rate (MHHOS) as a damping controller for power system oscillations. Yousri et al. (2020) designed a modified HHO with the biological features of the rabbit (MHHO) for optimal photovoltaic array reconfiguration.

(2) Hybridization with other algorithms: Hybridizing HHO with other algorithms enables the advantages of two algorithms to be combined and their shortcomings overcome. This is another method for enhancing HHO's performance. Kamboj et al. (2020) suggested a hybrid algorithm called hHHO-SCA using a sine cosine algorithm, in which the SCA is employed to keep the exploration and exploitation phases balanced. Dhawale and Kamboj (2020) proposed a hybridized HHO with enhanced gray wolf optimization (IGWO) to boost population diversity and convergence. Zhong et al. (2020) hybridized HHO with the first-order reliability (FORM), and the HHO-FORM method was developed to extract the global optimal solutions for problems with high dimensions. Yıldız et al. (2019) proposed the hybridization HHO with Nelder-Mead called H-HHONM, in which the process parameters in milling operations are successfully optimized. A hybrid algorithm, termed hybrid HHO with simulated annealing algorithm (HHOSA), was introduced by Kurtuluş et al. (2020) to accelerate the convergence speed. SA is added into the hawks' phase to increase the convergence pace of the process. Fu et al. (2020a) proposed the hybridization HHO with a mutation sine cosine algorithm (MSCA) to diagnose the faults in rolling bearings. Aiming at accelerating HHO's exploitation, Fu et al. (2020b) proposed the hybridization HHO with mutation-based GWO (MHHOGWO), in which mutation-based GWO can improve the property of HHO. Bao et al. (2019) presented a hybrid HHO (HHO-DE) with a differential evolution algorithm (DE), where the DE is used to augment the required global and local search capabilities. Abd Elaziz et al. (2020) proposed a hybrid HHO (HHOSSA) with the salp swarm method (SSA) for picture segmentation issues to boost population diversity and improve the convergence rate. An enhanced hybrid version of fireworks method and HHO together with dynamic competition idea has been proposed in Li W. et al. (2021).

Although a large number of research work has paid attention to the HHO, some room for improvement still exists, especially when the algorithm is used to solve some new application scenarios. On the one hand, just like the no free lunch (Wolpert and Macready, 1997) that no one universal algorithm can solve all existing optimization problems, which demonstrates that no one can build an all-time-best-performing algorithm capable of solving all optimization problems. This means that while some algorithms excel at solving a subset of problems, they cannot guarantee the success of all optimization tasks involving diverse or complex scenes. Addressing this mindset, new optimization approaches or modified versions of existing techniques should be provided in the future to tackle subgroups of challenges in many disciplines. In addition, HHO is frequently plagued by inadequate exploitation and delayed convergence (Alabool et al., 2021), and the possibility of local optimal stagnation also exists for some multimodal or complex numerical optimization tasks. In this article, we propose a framework for fireworks explosion-based Harris Hawks optimization (FWHHO), which incorporates fireworks explosion search into the HHO algorithm for improving its performance on complex optimization problems. To the best of our knowledge, fireworks explosion is introduced into HHO for the first time in all the literature.

## 3. Proposed FWHHO algorithm

### 3.1. Basic HHO

Heidari et al. (2019) created the Harris Hawks optimization (HHO), a swarm-based optimization technique. HHO's primary objective is to imitate hawk's team coordination and prey escape in nature to develop answers to the single-objective problem. The HHO model consisted of three major stages: the exploration phase, the transition from exploring to exploiting, and the exploitation phase. These three stages are explained in the next few sections.

#### 3.1.1. Execution exploration

In this part, hawks detect prey in exploring by Equation 1; first, a population *X*_*i*_ (*i* = 1, 2, 3, 4…*N*) is randomly generated, where *N* is the number of hawks. When *q* ≥ 0.5 (a random value in [0, 1]), *X*_*i*_(*t* + 1) = *X*_rand_(*t*) − *r*_1_|*X*_rand_(*t*) − 2*r*_2_*X*(*t*)|; *X*_*i*_(*t* + 1) = (*X*_prey_(*t*) − *X*_m_(*t*) − *Y*), when *q* < 0.5. These two ways describe how hawks identify prey using a random perch and the true individual's position, respectively.


(1) $$X_{i}(t+1)=\{\begin{array}{ll} X_{\text{rand }}(t)-r_{1}\left|X_{\text{rand }}(t)-2r_{2}X(t)\right|, & q\geq 0.5\\ \left(X_{\text{prey }}(t)-X_{\text{m}}(t)-Y\right), & q<0.5 \end{array}$$

Where *X*_*i*_(*t* + 1) is the *i*^*th*^ new position of hawks in the (*t* + 1)^*th*^ iteration. *X*_rand_(*t*) is the *i*^*th*^ position of hawks in the *t*^*th*^ iteration, *r*_1_, *r*_2_, *r*_3_, and *r*_4_ are all random values in [0, 1]. *X*_prey_(*t*) is the current prey position in the *t*^*th*^ iteration. *X*_m_(*t*) is calculated by *Y* = *r*_3_(Lb + *r*_4_(Ub − Lb)) and reflects the difference between variables' upper and lower limits.

#### 3.1.2. Execution from exploration to exploitation

The escaping energy (*E*) controls the behavior of hawks from exploration to exploitation. The *E* is calculated by *E* = 2*E*_0_(1 − (*t*/*T*)), where *E*_0_ is the initial energy of the prey that randomly changes in (–1, 1). If |*E*| ≥ 1, the exploration phase will still execute; if |*E*| < 1, the exploitation phase is activated.

#### 3.1.3. Execution exploitation

This phase seeks to simulate the surprise pounce (seven kills) behavior of the hawk on the examined target. Four chasing tactics are offered to do this, namely (1) soft besiege, (2) hard besiege, (3) soft besiege with progressive rapid dives, and (4) hard besiege with progressive rapid dives.

**Soft besiege** If |*E*| ≥ 0.5 and *r* ≥ 0.5, soft besiege is activated. This behavior is modeled as *X*_*i*_(*t* + 1) = Δ*X*(*t*) − *E*|JX_prey_(*t*) − *X*(*t*)|, where Δ*X*(*t*) is calculated as Δ*X*(*t*) = *X*_prey_(*t*) − ∣*X*(*t*), Δ*X*(*t*) is the difference between the rabbit's position vector and its current location in the *t*^*th*^ iteration. *U* = 2(1 − *r*_5_) strategy for evading prey that varied randomly in each repetition. *r*_5_ is a random value in [0, 1].

**Hard besiege** Hard besiege is activated when |*E*| < 0.5 and *r* ≥ 0.5. This signifies that the victim is unable to flee successfully due to exhaustion. The new positions of hawks can be obtained by *X*(*t* + 1) = *X*_prey_(*t*) − *E*|Δ*X*(*t*)|.

**Soft besiege with progressive rapid dives** Soft besiege with progressive rapid dives is activated when |*E*| ≥ 0.5 and *r* < 0.5. Hawks must then choose the optimal dive angle toward the prey in this situation by evaluating the new moves using *Y* = *X*_prey_(*t*) − *E*|*JX*_prey_(*t*) − *X*(*t*)|. If the comparison result does not result in determining the best dive toward the prey, team rapid dives based on the levy flight *LF* are performed to improve the exploitation capacity as modeled by *Z* = *Y* + *S*× LF(*D*), where *D* is the number of dimensions, *S* represents random vector by size 1 × *D*, and *LF* is calculated as follows:


(2) $$\mathrm{LF}(X)=\frac{u\times \sigma }{|v{|}^{\frac{1}{\beta }}},\sigma ={\left(\frac{\text{Γ}(1+\beta )\times \sin\left(\frac{\pi \beta }{2}\right)}{\text{Γ}\left(\frac{1+\beta }{2}\right)\times \beta \times 2^{\left(\frac{\beta -1}{2}\right)}}\right)}^{\frac{1}{\beta }}$$

Where *u* and *v* represent random values in [0, 1]. *B* is a constant with 1.5. Therefore, in this way, the new positions *X*(*t* + 1) of hawks can be calculated as *X*(*t* + 1) = *Y* if *F*(*Y*) < *F*(*X*(*t*)); *X*(*t* + 1) = *Z* if *F*(*Z*) < *F*(*X*(*t*)), where *F* is a fitness function for an optimization problem.

**Hard besiege with progressive rapid dives** In this strategy, prey had no energy to escape |*E*| < 0.5, and hawks constructed hard besiege *r* < 0.5. The new positions *X*(*t* + 1) of hawks can be calculated as *X*(*t* + 1) = *Y* if *F*(*Y′*) < *F*(*X*(*t*)), *X*(*t* + 1) = *Z* if *F*(*Z′*) < *F*(*X*(*t*)), where *Y′* = *X*_prey_(*t*) − *E*|*JX*_prey_(*t*) − *X*_m_(*t*)|, and *Z′* = *Y′* + *S* × LF(*D*). The distinction between this strategy and the previous one (soft besiege with progressive rapid dives) is that the hawks are striving to reduce the average distance between their location and their prey.

### 3.2. FWHHO framework

HHO is an excellent prospector but a poor exploitation candidate, although it has several local search mechanisms. However, an efficient search process demands a high level of exploration to discover prospective solutions inside the board search space, as well as a high level of exploitation to further increase the attributes of those solutions. Tan and Zhu (2010) recently presented a fireworks algorithm (FWA). The primary FWA operator is fireworks explosion search, which results in the creation of new persons in the vicinity of a few potential individuals. This operator can efficiently use data to find better solutions (Zhang et al., 2014). A novel hybrid FWHHO framework will be proposed in this research, which incorporates the fireworks explosion search. The FWHHO framework generates an initial population of N and then progresses through four search phases: (1) exploration, (2) transition from exploration to exploitation, (3) exploitation, and (4) fireworks explosion search. The first three phases are formed by HHO, whereas the fourth phase is formed by FWA.

#### 3.2.1. Fireworks algorithm

Following three levels of hawk search, a small number of individuals are chosen to do the fireworks explosion search. The best candidate is chosen first, followed by those who are closest to the top candidate. The following formula is used to calculate the distance between two food individuals as $R\left(X_{i}\right)=\underset{j\in K}{\sum }d\left(X_{i},X_{j}\right)=\underset{j\in K}{\sum }||X_{i}-X_{j}||$, where ***X***_*i*_ and ***X***_*j*_(*j* ≠ *i*) are the different individuals, and *K* is the number of individuals. The chance of an individual being selected for a search for fireworks explosions is calculated by *p*(*X*_*i*_) = *R*(*X*_*i*_)/∑_*j*∈*K*_*R*(*X*_*j*_). When a spark explodes, several sparks emerge around it, generating new people. The operator also has two parameters to be determined. The number of sparks *S*_*i*_ is calculated as follows:


(3) $$S_{i}=\hat{S}\cdot \frac{Y_{\max}-f\left(X_{i}\right)+\varepsilon }{\sum_{i=1}^{ke}\left(Y_{\max}-f\left(X_{i}\right)\right)}$$

Where the *i*^*th*^ individual generates the *S*_*i*_ sparks, the number of sparks is Ŝ, and the total number of people chosen for the explosion search is *ke*. *Y*_max_ is the best individual. *f*(***X***_*i*_) is the *i*^*th*^ individual. The amount of sparks is restricted by an upper constraint *S*_*max*_ and a lower bound *S*_*min*_ in order to avoid overpowering impacts of pyrotechnics in desirable locations. If *S*_*i*_ < *S*_min_, *S*_*i*_ = *S*_min_, then *S*_*i*_ > *S*_max_, *S*_*i*_ = *S*_max_.

The second parameter is the amplitude of the generated sparks $A_{i}^{g}$. The magnitude of the explosion is dynamically adjusted. If *g* = 1, $A_{i}^{g}=A_{i}^{1}$, then $f\left(V_{\text{best}}^{\text{g}}\right)\geq f\left(X_{i}^{\text{g}}\right)$, $A_{i}^{g}=C_{r}A_{i}^{g-1}$, $A_{i}^{g}=C_{a}A_{i}^{g-1}$ when $f\left(V_{\text{best}}^{\text{g}}\right)<f\left(X_{i}^{\text{g}}\right)$, where $A_{i}^{g}$ denotes the amplitude of the *i*^*th*^ individual's explosion, while the diameter of the search zone is denoted by the first generation's amplitude. $V_{\text{best}}^{g}$ is the best spark created, while *g* denotes the generation count. If the objective function value of the best spark is greater than the value of the individual, the amplitude multiplies with a coefficient *C*_*a*_ > 1; otherwise, it multiplies with a coefficient *C*_*a*_ < 1.

In this case, the amplitude is too small to make more progress and should be increased; if a better solution is not found, it means that the amplitude is too long and should be cut down. The dynamic amplitude can be used to make more space around possible solutions.

#### 3.2.2. Proposed FWHHO

The FWHHO is intended to overcome the inherent flaws of the original HHO through inefficient exploitation and slow convergence. The fireworks algorithm's explosive search mechanism is included in HHO in this research, along with a framework for fireworks explosion-based Harris Hawks optimization (FWHHO). More precisely, the suggested FWHHO structure is divided into two search stages: Harris Hawks and fireworks explosion. A search for fireworks explosions is conducted to identify suitable places for developing improved hawk solutions. To the best of our knowledge, this is the first time that the HHO is integrated with operators of fireworks explosions. In the proposed FWHHO, three sections are available for discussion. The first step is to carry out the initialization of the search population in the database. The second step is to complete the execution of the algorithm operators in the original HHO, and the third step is to complete the execution of the newly introduced operators generated from the fireworks explosion that was introduced earlier. Algorithm 1 presents the pseudocode for the FWHHO algorithm utilizing the basic HHO.

**Algorithm 1:** FWHHO.

** 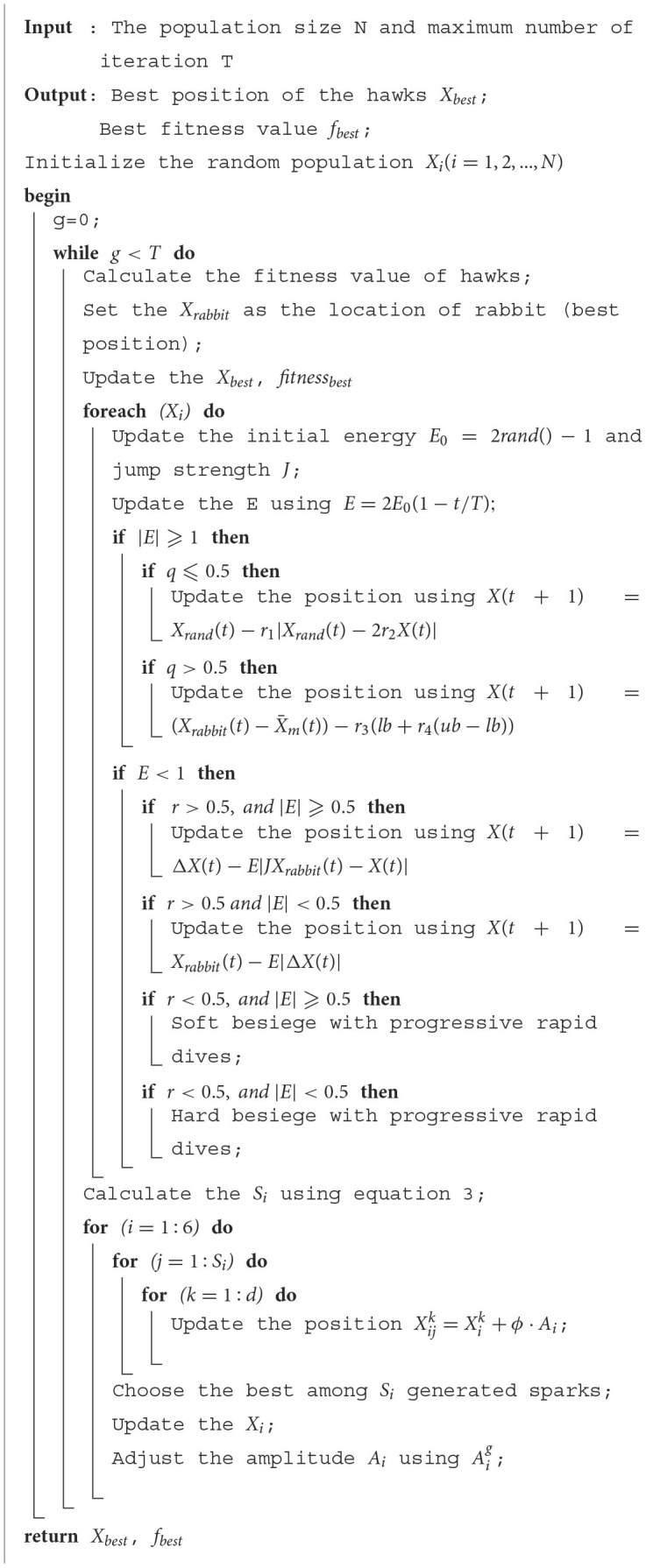 **

## 4. Experimental designs

In this study, some experiments are executed to verify the proposed FWHHO, which is composed of a comparison between FWHHO and state-of-the-art algorithms, a comparison of FWHHO on existing HHO algorithms, a comparison between FWHHO and fireworks algorithms, and an application of FWHHO on machine learning evolution. For comparison between FWHHO and state-of-the-art algorithms, existing HHO algorithms, fireworks algorithms, and the CEC2014 benchmark functions (Liang et al., 2013) are used which has 30 functions including F1–F3 (unimodal functions), F4–F16 (simple multimodal functions), F17–F22 (hybrid functions), and F23–F30 (composition functions). The CEC2014 benchmark has the most typical and comprehensive test function and can effectively test the performance of the algorithm. The maximum number of function evaluations is 5*10^4^. To record the statistical data, each algorithm is run 30 times independently on each function. For the application of FWHHO on machine learning evolution, the key parameters and optimal subfeatures of the support vector machine on medical diagnosis. Take note that all tests are run on Windows Server 2018 R2 with MATLAB2020b on a Xeon CPU i5-2660 V3 (2.60 GHz) and 16 GB RAM.

## 5. Experimental and analytical results

### 5.1. Comparison between FWHHO and state-of-the-art algorithms

In this section, the proposed FWHHO is compared to several state-of-the-art algorithms on CEC 2014 benchmarks and each algorithm is carried out 30 times independently. These state-of-the-art algorithms are composed of original HHO (Heidari et al., 2019), self-adaptive differential evolution (SaDE) (Qin et al., 2008), comprehensive learning particle swarm optimization (CLPSO) (Liang et al., 2006), gravitational search algorithm (GSA) (Rashedi et al., 2009), gray wolf optimizer (GWO) (Mirjalili et al., 2014), sine cosine algorithm (SCA) (Mirjalili, 2016), and salp swarm algorithm (SSA) (Mirjalili et al., 2017). These algorithms' parameters have been set in accordance with the original article. As shown in Table 1, the mean and standard deviation (STD) values demonstrate the accuracy of the experimental findings.

**Table 1:** Comparison of FWHHO and state-of-the-art algorithms.

**Algorithm**	**F1**		**F2**		**F3**		**F4**		**F5**	
	**Mean**	**STD**	**mean**	**STD**	**Mean**	**STD**	**Mean**	**STD**	**mean**	**STD**
FWHHO	**902167.7**	**341849.9**	**3721.089**	**4405.924245**	**4673.31**	**1982.922**	**489.6231**	**30.78631**	520.62336	0.1982208
HHO	84,204,281	29,097,119	344,128,875	89744199.45	86829.654	17975.939	860.59266	153.66426	520.98054	0.1139413
SaDE	13,660,679	6,116,815	411873.72	881877.5426	12802.05	4032.3343	571.68022	31.14679	520.94086	0.0482358
CLPSO	80,622,496	19,279,303	14,519,980	5654419.135	14977.062	3239.0141	691.03257	43.910697	520.65163	0.0311069
GSA	9901563.4	2741841.8	21,871,458	2877324.194	158289.23	19851.048	566.46908	53.632445	521.20296	0.0275089
GWO	152,581,247	80,818,647	1.505E+10	5,279,434,518	86938.199	14549.639	2004.5215	563.8034	521.21368	0.0333388
SCA	1.083E+09	300,773,742	6.804E+10	6,253,978,317	121746.57	9788.8131	11416.512	1707.6495	521.16751	0.0426289
SSA	19,120,011	6525574.8	7211.5226	8780.226044	91301.134	26719.579	528.5813	31.661059	**520.0749**	**0.122223**
**Algorithm**	**F6**		**F7**		**F8**		**F9**		**F10**	
	**Mean**	**STD**	**Mean**	**STD**	**Mean**	**STD**	**Mean**	**STD**	**Mean**	**STD**
FWHHO	**622.5629**	**3.238746**	700.01446	0.019920524	**801.0945**	**1.441833**	**1025.692**	**18.94712**	1156.0303	157.76935
HHO	661.61438	4.0947947	705.22059	1.232503654	1100.7096	17.165175	1313.713	54.03385	7606.5904	1164.6075
SaDE	631.84938	3.9399137	700.303	0.468911669	827.95886	10.270008	1029.5277	27.745652	1132.1985	84.613587
CLPSO	643.09069	1.8463397	701.1039	0.063808641	804.3186	2.4776972	1155.414	16.847765	**1085.303**	**57.57045**
GSA	634.55438	4.1153616	701.19855	0.036468763	1016.2846	24.572562	1158.2949	21.128392	6045.4593	765.49163
GWO	636.97537	3.5741224	796.62806	45.37275951	1049.0515	46.231238	1142.0906	78.524089	7345.8181	696.9429
SCA	671.08537	3.0570904	1376.1867	102.3210958	1351.9898	34.304098	1521.8356	37.488102	13743.116	574.61923
SSA	646.87416	5.9129457	**700.005**	**0.005319901**	1093.5225	65.435431	1223.6621	69.487215	8237.9232	1427.6469
**Algorithm**	**F11**		**F12**		**F13**		**F14**		**F15**	
	**Mean**	**STD**	**Mean**	**STD**	**Mean**	**STD**	**Mean**	**STD**	**Mean**	**STD**
FWHHO	**6246.174**	**426.2649**	**1200.54**	**0.221840141**	1300.4953	0.0606232	**1400.319**	**0.026883**	**1522.16**	**3.401352**
HHO	11019.188	1009.8149	1202.8878	0.582795775	1300.6048	0.0822885	1400.3935	0.1112097	1612.2536	17.092755
SaDE	11703.602	481.11301	1201.8885	0.201954325	1300.5817	0.0741299	1400.3503	0.046948	1557.7386	17.321341
CLPSO	8580.4431	379.44062	1200.7165	0.090524689	1300.4352	0.0613921	1400.3504	0.0382548	1544.6994	4.6867086
GSA	7017.7047	392.05419	1200.5767	0.081645677	**1300.389**	**0.049106**	1400.3403	0.0383133	1522.8408	1.3660223
GWO	8697.2593	3045.7388	1203.5457	1.040535643	1301.424	1.227591	1430.3369	15.090128	14624.782	14740.197
SCA	15254.287	317.06646	1204.0751	0.339168547	1305.2589	0.4376067	1562.6307	18.245607	382716.45	189109.34
SSA	7670.6698	642.03634	1201.2851	0.481616691	1300.6868	0.1214973	1400.4498	0.2479282	1531.9912	9.2806381
**Algorithm**	**F16**		**F17**		**F18**		**F19**		**F20**	
	**Mean**	**STD**	**Mean**	**STD**	**Mean**	**STD**	**Mean**	**STD**	**Mean**	**STD**
FWHHO	**1619.428**	**0.345012**	**66481.84**	**47638.15479**	3088.449	1266.4386	1943.658	**17.10113**	**15142.42**	**12817.1**
HHO	1622.4671	0.413234	18460345	7972578.739	13,710,469	34,419,944	1981.637	27.02821	55128.152	11374.184
SaDE	1621.6403	0.1870237	1152268.7	605833.0556	**2872.002**	**939.0795**	1954.4916	26.592052	23357.606	8746.3568
CLPSO	1620.5242	0.4427087	17,848,523	9532081.885	154858.91	143572.05	1965.3165	12.963449	28425.059	9727.8192
GSA	1622.9664	0.3687285	1718458.1	618217.1251	646399.74	135414.25	**1942.6324**	30.551672	125093.16	48852.004
GWO	1621.1964	0.9847289	8114432.6	5066114.344	278,141,581	385,322,789	2047.2492	61.782907	32954.921	12503.212
SCA	1622.8699	0.3294154	72,844,730	25410304.55	2.394E+09	630,097,903	2318.4619	84.557359	54164.871	14916.239
SSA	1621.4467	0.4273696	1414872.1	847605.5879	3447.7937	1281.8608	1965.1462	26.355587	38407.844	22540.267
FWHHO	**806869.9**	**2,395,752**	3164.8182	183.1421049	**2,500**	**0**	2686.9589	5.8057083	**2,700**	**0**
HHO	6971504.4	6238150.4	3991.3549	398.0434698	2644.0045	1.462E-12	2600.0001	0.0003461	2703.907	4.419675
SaDE	1145810.5	443279.41	**2953.843**	**327.8212563**	2644.3647	0.7882839	2687.2156	5.2903316	2729.9284	3.0508245
CLPSO	8306111.8	3406855.5	3143.8321	275.459617	2649.3902	1.3461726	2672.6421	2.567531	2728.2043	3.2505201
GSA	1,768,386	867117.77	3868.3596	277.0093698	2649.2015	0.8835197	2644.0439	20.135419	2729.2335	13.198939
GWO	2308013.2	2499719.1	3274.8755	319.4672753	2809.3229	84.003253	**2600.023**	**0.008835**	2726.5001	14.163469
SCA	16338955	8169734.2	5000.4361	294.5817911	3128.0566	86.118664	2691.4821	61.788571	2764.4036	42.596036
SSA	1599820.7	778936.99	3474.514	291.412842	2669.6498	8.6901173	2699.6735	5.3489669	2738.5007	7.8465751
**Algorithm**	**F26**		**F27**		**F28**		**F29**		**F30**	
	**Mean**	**STD**	**Mean**	**STD**	**Mean**	**STD**	**Mean**	**STD**	**Mean**	**STD**
FWHHO	2721.1193	41.651775	**2,900**	**4.79E-13**	**3,000**	**4.79E-13**	**3,100**	**0**	**14426.37**	**1466.246**
HHO	2790.0503	31.463626	3672.3892	3672.389231	66.423926	4420.6095	287.9402	4428.6234	183.17187	72763.522
SaDE	2790.4146	31.548118	3765.7075	76.90338617	4511.6262	118.40447	18094.403	41410.012	20598.228	3738.187
CLPSO	2712.3271	32.125417	4054.9582	250.846698	5030.3248	481.3801	105417.03	33546.662	27081.575	4087.2807
GSA	2800.4487	0.0926686	4691.0312	1350.850628	10075.937	872.97659	20558298	64744528	88885.678	28741.379
GWO	2800.2059	0.3530995	3906.3114	110.5318484	6266.9374	695.97127	32,503,381	30,568,058	484817.75	245248.7
SCA	2705.9092	0.4627085	4884.8992	97.55160642	9729.4812	615.86949	30,6298,490	41364554	3335276.6	1058545.4
SSA	**2700.686**	**0.097619**	4256.8142	169.4583249	5073.2733	728.69706	56,708,696	44,745,741	94378.038	41880.728

Where bold indicates that the current value is the best in all algorithms.

Even if it may not perform well in all circumstances, it can be seen that the proposed FWHHO has the best average value performance among most benchmarks. To establish how significant the gains were, the Wilcoxon sign rank test was also utilized. *P* < 0.05 show that FWHHO works better than the other optimizer. In this case, the gains are not statistically significant. Table 2 contains the calculated p-values. *P* > 0.05 are highlighted in Table 2, indicating that the difference is not statistically significant. The calculated *p*-values of FWHHO and other state-of-the-art algorithms on CEC2014 benchmarks are shown in Table 2. As shown in Table 2, most of the *p* < 0.05, demonstrating that FWHHO outperformed the original HHO and other state-of-the-art algorithms.

**Table 2:** The calculated p-values for FWHHO vs. state-of-the-art algorithms.

**F**	**HHO**	**SaDE**	**CLPSO**	**GSA**	**GWO**	**SCA**	**SSA**
F1	0.001953125+	0.001953125+	0.001953125+	0.001953125+	0.001953125+	0.001953125+	0.001953125+
F2	0.001953125+	0.083984375+	0.001953125+	0.001953125+	0.001953125+	0.001953125+	0.043164062+
F3	0.001953125+	0.001953125+	0.001953125+	0.001953125+	0.001953125+	0.001953125+	0.001953125+
F4	0.001953125+	0.00390625+	0.001953125+	0.005859375+	0.001953125+	0.001953125+	0.037109375+
F5	0.005859375+	0.005859375+	0.001308593+	0.001953125+	0.00390625+	0.001953125+	0.061953125-
F6	0.001953125+	0.001953125+	0.001953125+	0.00390625+	0.001953125+	0.001953125+	0.001953125+
F7	0.001953125+	0.001953125+	0.001953125+	0.001953125+	0.001953125+	0.001953125+	0.431640625-
F8	0.001953125+	0.001953125+	0.01953125+	0.001953125+	0.001953125+	0.001953125+	0.001953125+
F9	0.001953125+	0.695312500–	0.001953125+	0.001953125+	0.001953125+	0.001953125+	0.001953125+
F10	0.001953125+	0.625000000-	0.193359375–	0.001953125+	0.001953125+	0.001953125+	0.001953125+
F11	0.001953125+	0.001953125+	0.001953125+	0.009765625+	0.009765625+	0.001953125+	0.001953125+
F12	0.001953125+	0.001953125+	0.064453125–	0.431640625–	0.001953125+	0.001953125+	0.001953125+
F13	0.009765625+	0.001953125+	0.048828125+	0.009765625+	0.001953125+	0.001953125+	0.005859375+
F14	0.009765625+	0.083984375+	0.105468750–	0.160156250–	0.001953125+	0.001953125+	0.130859375–
F15	0.001953125+	0.001953125+	0.001953125+	0.6953125+	0.001953125+	0.001953125+	0.02734375+
F16	0.001953125+	0.001953125+	0.00390625+	0.001953125+	0.001953125+	0.001953125+	0.001953125+
F17	0.001953125+	0.001953125+	0.001953125+	0.001953125+	0.001953125+	0.001953125+	0.001953125+
F18	0.001953125+	0.431640625–	0.001953125+	0.001953125+	0.001953125+	0.001953125+	0.625000000-
F19	0.02734375+	0.232421875–	0.01953125+	1–	0.001953125+	0.001953125+	0.009765625+
F20	0.001953125+	0.193359375+	0.048828125+	0.001953125+	0.02734375+	0.001953125+	0.00390625+
F21	0.013671875+	0.083984375+	0.00390625+	0.033984375+	0.044453125+	0.00390625+	0.083984375+
F22	0.001953125+	0.064453125-	0.556640625-	0.001953125+	0.275390625–	0.001953125+	0.037109375+
F23	0.001953125+	0.001953125+	0.001953125+	0.001953125+	0.001953125+	0.001953125+	0.001953125+
F24	0.001953125+	0.845703125–	0.001953125+	0.001953125+	0.001953125+	0.921875–	0.00390625+
F25	0.001953125+	0.001953125+	0.001953125+	0.001953125+	0.001953125+	0.037109375+	0.001953125+
F26	0.02734375+	0.013671875+	0.232421875–	0.001953125+	0.005859375+	0.431640625–	0.193359375–
F27	0.001953125+	0.01953125+	0.005859375+	0.009765625+	0.00390625+	0.001953125+	0.001953125+
F28	0.001953125+	0.032226562+	0.00390625+	0.001953125+	0.001953125+	0.001953125+	0.001953125+
F29	0.001953125+	0.02734375+	0.001953125+	0.001953125+	0.001953125+	0.001953125+	0.001953125+
F30	0.083984375+	0.001953125+	0.001953125+	0.001953125+	0.001953125+	0.001953125+	0.001953125+

**Figure 1:**
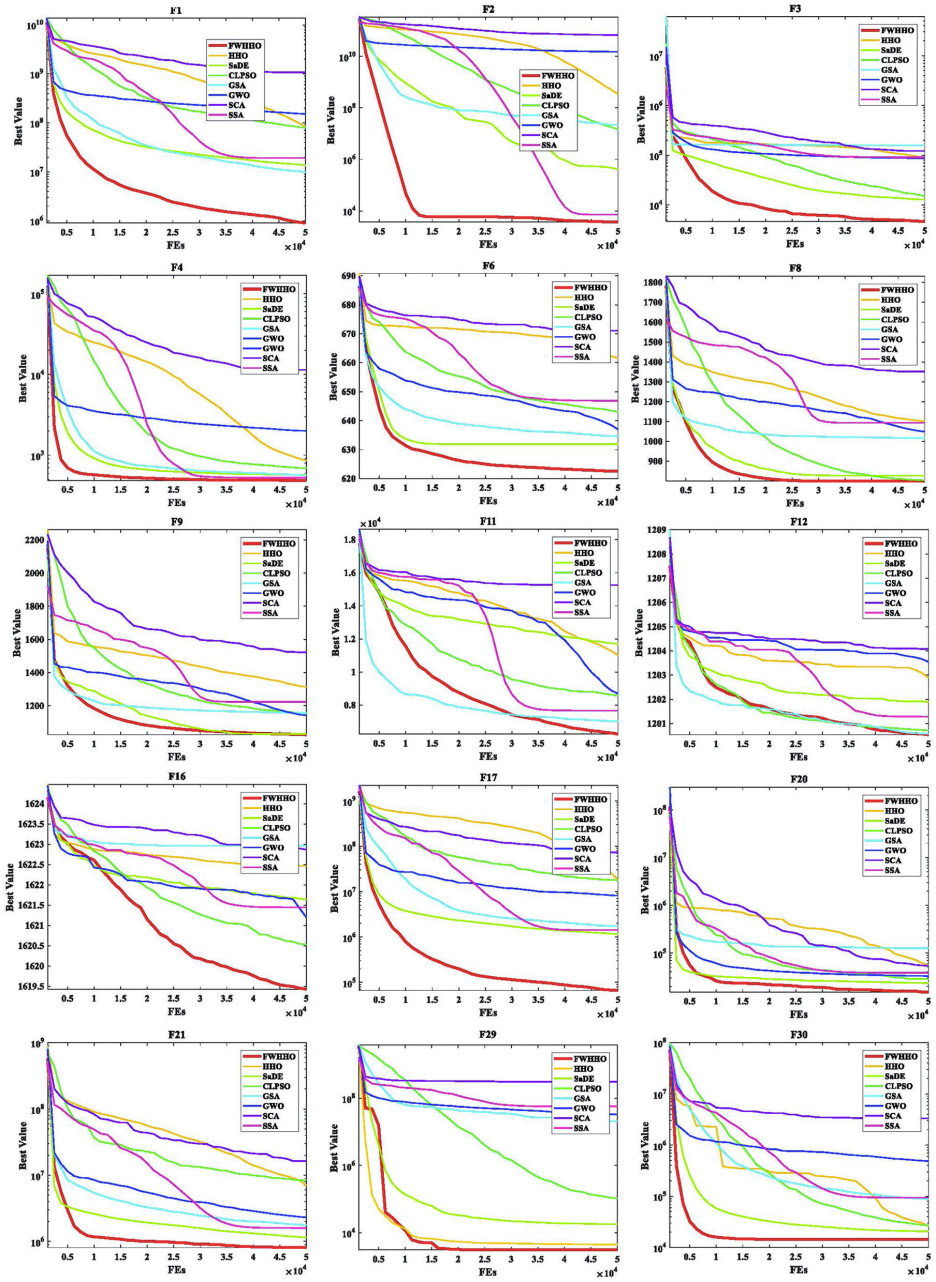
Convergence curves of FWHHO and state-of-the-art algorithms.

Additionally, Figure 1 illustrates the convergence curves of various algorithms for a selection of benchmarks. It can be observed that the presented FWHHO has a fast search ability of convergence on most benchmarks such as F2, F3, F4, F8 F20, and F30, to ensure that it achieves the best theoretical value in a short time and the apparent superiority to all other competitors in these benchmarks. Convergence can occur quickly during the early stages of algorithm execution, particularly for F4, F29, and F30. Although the convergence ability of F11, F12, and F16 are relatively weak in the early stages of algorithm execution, as the number of iterations rises, it can achieve quick convergence in the later stages. In a nutshell, it can be stated that the original HHO's properties can be significantly enhanced.

### 5.2. Comparison between FWHHO and existing HHO algorithms

On the CEC2014 benchmarks, this section compares the proposed FWHHO and existing HHO algorithms. The existing HHO algorithms are composed of CMDHHO (enriched HHO with chaos strategy, multi-population mechanism, and DE strategy) (Chen et al., 2020a), hHHO-SCA (hybrid harris hawks-sine cosine algorithm) (Kamboj et al., 2020), EHHO (enriched Harris Hawks optimization with chaotic drifts) (Chen et al., 2020b), and GCHHO (HHO with Gaussian mutation and cuckoo search strategy) (Song et al., 2021). All of these algorithms' parameters have been set in accordance with the original article. Table 3 compares the performance of FWHHO and existing HHO algorithms on the CEC2014 benchmark with 50 dimensions. As can be shown, FWHHO obviously surpasses these current HHO algorithms in terms of not only mean error values but also standard deviations for the majority of functions, while FWHHO may perform badly in contrast to other existing FWA algorithms for F19, F21, F24, and F30. On F19, the GCHHO produces the best mean error values, while the hHHO-SCA produces the best mean error values. On F24 and F30, the CMDHHO obtains the best mean error values.

**Table 3:** Comparison of FWHHO and existing HHO algorithms.

**Algorithm**	**F1**		**F2**		**F3**		**F4**		**F5**	
	**Mean**	**STD**	**Mean**	**STD**	**Mean**	**STD**	**Mean**	**STD**	**Mean**	**STD**
FWHHO	**902167.6951**	**341849.9335**	**3721.088889**	**4405.924245**	**4673.310063**	**1982.921821**	**489.6231251**	**30.78630585**	**520.6233586**	**0.198220769**
CMDHHO	103890312.8	33001287.88	327793744.7	92926014.92	85451.81297	9105.141855	782.6948035	103.6429363	521.0630324	0.058730331
hHHO-SCA	4569048.312	1805547.978	247988.9419	198675.7211	107520.0623	48576.35026	522.7629243	62.06323585	521.2452445	0.024439631
EHHO	796054016.9	113181710.4	37,567,585,329	2,049,620,919	149739.7534	14364.36915	4233.704968	961.1239732	521.2266988	0.034571179
GCHHO	36189630.34	11491261.25	523691072.2	26865442.31	30421.48815	6186.703675	550.2216471	63.05463252	521.2059497	0.03939306
**Algorithm**	**F6**		**F7**		**F8**		**F9**		**F10**	
	**Mean**	**STD**	**Mean**	**STD**	**Mean**	**STD**	**Mean**	**STD**	**Mean**	**STD**
FWHHO	**622.5628793**	**3.238746129**	**700.014456**	**0.019920524**	**801.094455**	**1.441832654**	**1025.692072**	**18.94711974**	**1156.030304**	**157.7693517**
CMDHHO	662.1826891	4.546226379	704.6515607	1.368692467	1100.939188	11.82789133	1332.893431	27.72105463	7538.169473	1332.90993
hHHO-SCA	664.1289123	4.894426973	700.2644196	0.202129738	1222.199612	69.97551889	1490.525512	110.9866227	8783.941911	912.6304135
EHHO	663.6634491	1.732825499	1059.60411	20.37671422	1257.69023	13.85470965	1429.495493	18.32096447	14536.49428	420.5037096
GCHHO	647.2027653	6.558662276	705.7578814	0.48232051	1217.123591	34.57489263	1426.653947	37.01036111	10164.22	936.2988401
**Algorithm**	**F11**		**F12**		**F13**		**F14**		**F15**	
	**Mean**	**STD**	**Mean**	**STD**	**Mean**	**STD**	**Mean**	**STD**	**Mean**	**STD**
FWHHO	**6246.174232**	**426.2649298**	**1200.540007**	**0.221840141**	**1300.495307**	**0.060623172**	**1400.318819**	**0.026883173**	**1522.160239**	**3.401352123**
CMDHHO	10127.53539	1181.826873	1202.85012	0.592117692	1300.554465	0.097169013	1400.386586	0.130900487	1624.778284	28.54466223
hHHO-SCA	8936.77265	811.9290814	1202.378989	0.637422362	1300.57458	0.127391662	1400.383145	0.157614633	1665.629549	60.49873062
EHHO	14884.80398	443.4802388	1203.917382	0.350332971	1303.798112	0.265225751	1498.59787	15.56062776	131592.5695	39478.98002
GCHHO	11942.62429	685.7700386	1204.080914	0.262313389	1300.677125	0.116171067	1400.460773	0.244266746	1539.512526	2.397143599
**Algorithm**	**F16**		**F17**		**F18**		**F19**		**F20**	
	**Mean**	**STD**	**Mean**	**STD**	**Mean**	**STD**	**Mean**	**STD**	**Mean**	**STD**
FWHHO	**1619.428379**	**0.3450123**	**66481.84461**	**47638.15479**	**3088.448968**	**1266.438588**	1943.657601	17.10112995	**15142.42127**	**12817.09723**
CMDHHO	1622.427536	0.292927417	20554897.81	12000187.58	6792821.297	4773775.583	1975.572543	28.50502352	67063.54008	28688.89699
hHHO-SCA	1623.270028	0.517229679	638960.3153	344331.7002	117390.684	45844.90914	1958.832054	31.33723187	50223.6564	29032.06522
EHHO	1622.864134	0.147043699	52863052.66	11524394	1704318808	362823928.9	2162.594009	20.65138199	118778.7141	37271.21262
GCHHO	1622.241844	0.410334227	3284262.451	1262191.018	17192603.52	4397520.549	**1940.754136**	**19.8719176**	12596.89942	5105.428091
**Algorithm**	**F21**		**F22**		**F23**		**F24**		**F25**	
	**Mean**	**STD**	**Mean**	**STD**	**Mean**	**STD**	**Mean**	**STD**	**Mean**	**STD**
FWHHO	806869.942	2395752.482	**3164.818187**	**183.1421049**	**2,500**	**0**	2686.958891	5.805708267	**2703.906636**	**4.419674612**
CMDHHO	6823584.462	3436758.543	4318.101155	294.6438341	**2500**	**0**	**2600.00003**	**9.44433E-05**	2700	0
hHHO-SCA	**455135.2098**	**229919.3437**	4206.171199	335.1585916	2647.636654	2.447936742	2763.658341	43.75568919	2762.999585	10.83580829
EHHO	23895662.43	6184196.17	4622.940174	245.7591414	3091.837281	54.98753232	2820.19276	14.85514332	2816.957749	11.85633931
GCHHO	1469901.29	448172.3061	3629.073204	222.8974308	2666.621666	10.96236159	2691.171601	10.01300934	2740.558678	4.373412794
FWHHO	**2721.119273**	**41.65177484**	**2,900**	**4.79E-13**	**3,000**	**4.79346E-13**	**3,100**	**0**	14426.36671	1466.245516
CMDHHO	2,800	0	2,900	4.79346E-13	3,000	4.79346E-13	3,100	0	**3,200**	**0**
hHHO-SCA	2788.734482	109.363826	5038.703057	187.0619575	8660.939983	2764.212468	429011518.5	278726392.8	1626566.369	4960256.662
EHHO	2803.874255	109.7133846	4573.653153	34.60237798	6156.475876	655.8127536	68674442.79	12207851.15	839806.5244	202142.3024
GCHHO	2802.670264	0.497716584	4270.769952	396.6431602	12616.9585	2336.202397	3160.672309	49.85277448	33944.09408	35992.31236

Where bold indicates that the current value is the best in all algorithms.

Table 4 presents the *p*-values for the FWHHO and other existing HHO algorithms on the CEC2014 benchmark with 50 dimensions. As can be seen, the majority of cases are smaller than 0.05, indicating that FWHHO outperforms CMDHHO, hHHO-SCA, EHHO, and GCHHO. A preliminary result is that the FWHHO approach is superior to other existing HHO methods in terms of numerical optimization potential. In addition, the convergence curves of FWHHO and other existing HHO methods for several selected benchmarks are exhibited in Figure 3. The proposed FWHHO shows the best fast convergence speed among all these existing HHO methods on these functions. The estimated optimal solution can be reached fast during the early stages of FWHHO execution; in contrast, other algorithms did not complete convergence until the end of the iteration number.

**Table 4:** The calculated *p*-values for FWHHO vs. existing HHO algorithms.

**F**	**CMDHHO**	**hHHO-SCA**	**EHHO**	**GCHHO**
F1	0.001953+	0.001953+	0.001953+	0.001953+
F2	0.001953+	0.001953+	0.001953+	0.001953+
F3	0.001953+	0.001953+	0.001953+	0.001953+
F4	0.001953+	0.625000-	0.001953+	0.001953+
F5	0.001953+	0.001953+	0.001953+	0.001953+
F6	0.001953+	0.001953+	0.001953+	0.001953+
F7	0.001953+	0.001953+	0.001953+	0.001953+
F8	0.001953+	0.001953+	0.001953+	0.001953+
F9	0.001953+	0.001953+	0.001953+	0.001953+
F10	0.001953+	0.001953+	0.001953+	0.001953+
F11	0.001953+	0.001953+	0.001953+	0.001953+
F12	0.001953+	0.001953+	0.001953+	0.001953+
F13	0.322265–	0.130859–	0.001953+	0.001953+
F14	0.3222656–	0.695312–	0.001953+	0.105468–
F15	0.001953+	0.001953+	0.001953+	0.003906+
F16	0.001953+	0.001953+	0.001953+	0.001953+
F17	0.001953+	0.083984–	0.001953+	0.001953+
F18	0.001953+	0.001953+	0.001953+	0.001953+
F19	0.037109+	0.160156–	0.001953+	0.769531–
F20	0.001953+	0.001953+	0.001953+	0.064453–
F21	0.001953+	0.083984–	0.001953+	0.083984–
F22	0.001953+	0.001953+	0.001953+	0.001953+
F23	0.001953+	0.001953+	0.001953+	0.001953+
F24	0.001953+	0.001953+	0.001953+	0.431640–
F25	0.001953+	0.001953+	0.001953+	0.009765+
F26	0.556640–	0.001953+	0.375000–	0.001953+
F27	0.001953+	0.001953+	0.001953+	0.003906+
F28	0.001953+	0.001953+	0.001953+	0.001953+
F29	0.001953+	0.005859+	0.001953+	0.001953+
F30	0.001953+	0.001953+	0.001953+	0.3222656–

### 5.3. Comparison between FWHHO and fireworks algorithms

The new FWHHO algorithm is compared to many current fireworks algorithms on CEC2014 benchmarks in this section. The extant fireworks algorithms include the original FWA, the BBFWA (bare bones FWA) (Li and Tan, 2018), the LoTFWA (loser-out tournament-based FWA) (Li and Tan, 2017), and the GFWA (Guide FWA) (Li et al., 2016). Table 5 illustrates how these algorithms' parameters are set. The results of the statistical comparison between FWHHO and existing FWA algorithms on the CEC2014 benchmark with 50 dimensions are shown in Table 6. As can be seen, the FWHHO approach delivers the most thorough results in terms of mean error values and standard deviations for the majority of functions, although it may perform badly in contrast to other current FWA algorithms for F5, F9, F19, and F20. It is worth noticing that the mean values and standard deviations for F5, F6, F12, and F16 for all methods are rather similar. LoTFWA outperforms FWHHO, BBFWA, and GFWA on the F5, F9, F19, and F20 functions and the proposed FWHHO on all other functions.

**Table 5:** Parameters for fireworks algorithms.

**Algorithm**	**Parameters**
FWA	*n* = 6, λ = 50, *a* = 0.04, *b* = 0.8, *A*_max_ = 40
BBFWA	*n* = 300, *C*_*a*_ = 1.2, *C*_*r*_ = 0.9
LoTFWA	*n* = 6, *C*_*a*_ = 1.2, *C*_*r*_ = 0.9, σ = 0.2, λ = 300
GFWA	*n* = 6, *C*_*a*_ = 1.2, *C*_*r*_ = 0.9, *A*_max_ = 40, λ = 100,
	*a* = 0.1, *b* = 1

**Table 6:** Comparison of FWHHO and fireworks algorithms.

**Algorithm**	**F1**		**F2**		**F3**		**F4**		**F5**	
	**Mean**	**STD**	**Mean**	**STD**	**Mean**	**STD**	**Mean**	**STD**	**Mean**	**STD**
FWHHO	**902167.695**	**341849.9335**	**3721.088889**	**4405.92425**	**4673.310063**	**1982.92182**	**489.623125**	**30.786306**	520.6234	0.198221
FWA	824445788	135438669.8	36049377664	2600658732	148644.9627	15159.299	860.592662	153.66426	521.2084	0.050939
BBFWA	28664317.5	6575107.597	499813711.6	60940130	28326.86593	5263.03265	571.680221	31.14679	521.2203	0.032717
LoTFWA	14284849.2	4066322.912	1256945.613	355131.028	15654.99661	4522.39898	691.032573	43.910697	**520.5816**	**0.103567**
GFWA	4367736.75	2410506.089	248899.356	209061.864	116530.037	35861.1324	566.469083	53.632445	521.2328	0.018748
**Algorithm**	**F6**		**F7**		**F8**		**F9**		**F10**	
	**Mean**	**STD**	**Mean**	**STD**	**Mean**	**STD**	**Mean**	**STD**	**Mean**	**STD**
FWHHO	**622.562879**	**3.238746129**	**700.014456**	**0.01992052**	**801.094455**	**1.44183265**	1025.69207	18.94712	**1156.03**	**157.7694**
FWA	662.976762	1.327404961	1053.936786	29.8405673	1261.307941	25.7592357	1419.04654	21.211026	14129.85	590.0503
BBFWA	645.863702	4.374185347	706.0391384	0.36358028	1216.546432	40.2947715	1431.90525	65.204238	10514.12	853.9626
LoTFWA	628.779375	3.471259522	700.8637366	0.06233911	1001.503205	39.5005997	**1106.17034**	**39.228711**	6992.855	825.6619
GFWA	663.799377	4.056487803	700.2913736	0.16120203	1238.674787	74.6237798	1486.16876	151.01315	8848.969	1059.42
**Algorithm**	**F11**		**F12**		**F13**		**F14**		**F15**	
	**Mean**	**STD**	**Mean**	**STD**	**Mean**	**STD**	**Mean**	**STD**	**Mean**	**STD**
FWHHO	**6246.17423**	**426.2649298**	**1200.540007**	**0.22184014**	**1300.495307**	**0.06062317**	**1400.31882**	**0.0268832**	**1522.16**	**3.401352**
FWA	14884.7458	358.8202421	1203.991823	0.51569947	1303.721196	0.2697012	1500.95821	8.273375	143772.6	26918.32
BBFWA	11795.6432	994.8879346	1203.79199	0.5153236	1300.634197	0.12095284	1400.61222	0.3449763	1540.428	2.484538
LoTFWA	6875.03748	1032.297375	1200.76176	0.38522606	1300.818638	0.1399847	1400.72899	0.4468956	1525.071	5.227916
GFWA	8704.30795	703.7929097	1202.128842	0.53278354	1300.507128	0.10599042	1400.33839	0.0480132	1678.261	56.35994
**Algorithm**	**F16**		**F17**		**F18**		**F19**		**F20**	
	**Mean**	**STD**	**Mean**	**STD**	**Mean**	**STD**	**Mean**	**STD**	**Mean**	**STD**
FWHHO	**1619.42838**	**0.3450123**	**66481.84461**	**47638.1548**	**3088.448968**	**1266.43859**	1943.6576	17.10113	15142.42	12817.1
FWA	1622.80144	0.274181665	50906676.49	14524703.6	1828889021	300873639	2149.44841	27.417311	114062.2	57835.91
BBFWA	1622.24163	0.438933308	2724565.658	984309.288	18561656.84	4827409.16	1933.42937	3.3285582	13240.95	3603.988
LoTFWA	1622.01211	0.443778625	1309160.404	581945.461	9863.774619	3158.81278	**1931.56337**	**14.29722**	**8073.586**	**3228.496**
GFWA	1623.27604	0.384993833	542569.3986	250678.937	124022.8052	69295.475	1968.80795	31.942968	35943.06	14838.63
**Algorithm**	**F21**		**F22**		**F23**		**F24**		**F25**	
	**Mean**	**STD**	**Mean**	**STD**	**Mean**	**STD**	**Mean**	**STD**	**Mean**	**STD**
FWHHO	**806869.942**	**2395752.482**	**3164.818187**	**183.142105**	**2500**	**0**	**2686.95889**	**5.8057083**	**2703.907**	**4.419675**
FWA	23835475.2	5317691.572	4635.747976	248.594987	3057.326898	76.427731	2818.35268	8.0137202	2817.509	19.72775
BBFWA	1203581.2	555428.1826	3763.902229	292.487265	2660.30961	6.05982363	2694.46262	7.7327575	2736.636	7.305577
LoTFWA	934271.314	661323.4123	3180.729589	237.184314	2661.397364	6.54233659	2687.69208	15.557818	2719.104	5.322087
GFWA	421879.706	235886.8118	4021.763343	386.111455	2647.557446	1.59247989	2760.55147	41.2113	2771.782	19.52749
FWHHO	**2721.11927**	**41.65177484**	**2900**	**4.79E-13**	**3000**	**4.7935E-13**	**3100**	**0**	**14426.37**	**1466.246**
FWA	2754.67909	82.14867568	4578.019934	71.9260213	6312.095084	929.167521	42638010.6	11926261	846007.9	158793.4
BBFWA	2823.32935	65.79974482	4397.418138	230.105296	13693.04679	1998.68032	88544.1495	269922.58	46481.42	55328.9
LoTFWA	2803.00271	94.06251765	3803.40262	152.563505	5046.212327	703.450337	69967.1801	37179.195	62375.13	45093.35
GFWA	2732.22495	47.30385255	4914.895686	163.639583	9628.899245	2349.15129	526,776,969	273,142,583	744237.7	2,144,963

Where bold indicates that the current value is the best in all algorithms.

**Figure 2:**
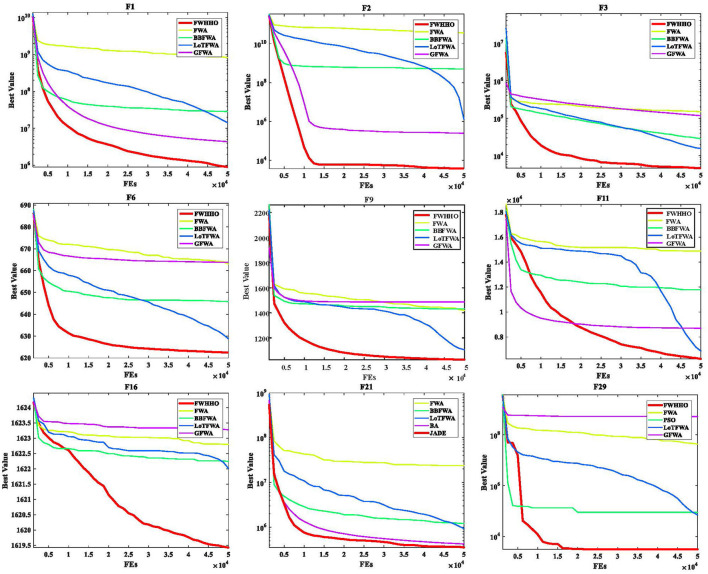
Convergence curves of FWHHO and existing HHO algorithms.

Table 7 presents the p-values for the FWHHO and other existing FWA algorithms on the CEC2014 benchmark with 50 dimensions. As can be seen, the majority of cases are smaller than 0.05, indicating that FWHHO considerably outperforms GFWA, BBFWA, LoTFWA, and FWA. A preliminary conclusion can be drawn that the algorithm proposed FWHHO in this study has the perfect potential ability for numerical optimization than other existing FWA methods. In addition, the convergence curves of FWHHO and other existing FWA methods for several selected benchmarks are exhibited in Figure 2. It can be observed that the suggested FWHHO has the fastest convergence speed of all the known FWA techniques on these functions. The approximate optimum solution may be promptly found in the early stage of FWHHO execution for F2, F21, and F29, but other algorithms did not complete the convergence until the end of the iteration number to acquire the approximate optimal solution. Overall, the technique suggested in this research has been first validated for several numerical optimization problems.

**Table 7:** The calculated p-values for FWHHO vs. fireworks algorithms.

**F**	**FWA**	**BBFWA**	**LoTFWA**	**GFWA**
F1	0.001953+	0.001953+	0.001953+	0.001953+
F2	0.001953+	0.001953+	0.001953+	0.001953+
F3	0.001953+	0.003906+	0.037109+	0.001953+
F4	0.001953+	0.003906+	0.003906+	0.003906+
F5	0.001953+	0.001953+	0.769531-	0.001953+
F6	0.001953+	0.001953+	0.013671+	0.001953+
F7	0.001953+	0.001953+	0.001953+	0.001953+
F8	0.001953+	0.001953+	0.001953+	0.001953+
F9	0.001953+	0.001953+	0.001953+	0.001953+
F10	0.001953+	0.001953+	0.001953+	0.001953+
F11	0.001953+	0.001953+	0.193359-	0.001953+
F12	0.001953+	0.001953+	0.083984-	0.001953+
F13	0.001953+	0.003906+	0.001953+	0.048828+
F14	0.001953+	0.1933593–	0.105468-	0.921875–
F15	0.001953+	0.003906+	0.625–	0.001953+
F16	0.001953+	0.001953+	0.001953+	0.001953+
F17	0.001953+	0.001953+	0.001953+	0.001953+
F18	0.001953+	0.001953+	0.001953+	0.001953+
F19	0.001953+	0.769531–	0.4921875–	0.01953+
F20	0.001953+	0.232421–	0.037109+	0.064453–
F21	0.001953+	0.064453+	0.083984-	0.083984–
F22	0.001953+	0.001953+	0.013671+	0.001953+
F23	0.001953+	0.001953+	0.001953+	0.001953+
F24	0.001953+	0.160156–	0.105468–	0.001953+
F25	0.001953+	0.009765+	0.005859+	0.001953+
F26	0.845703–	0.001953+	0.105468–	0.625–
F27	0.001953+	0.001953+	0.048828+	0.001953+
F28	0.001953+	0.001953+	0.027343+	0.001953+
F29	0.001953+	0.083984–	0.001953+	0.001953+
F30	0.001953+	0.130859–	0.001953+	0.001953+

**Figure 3:**
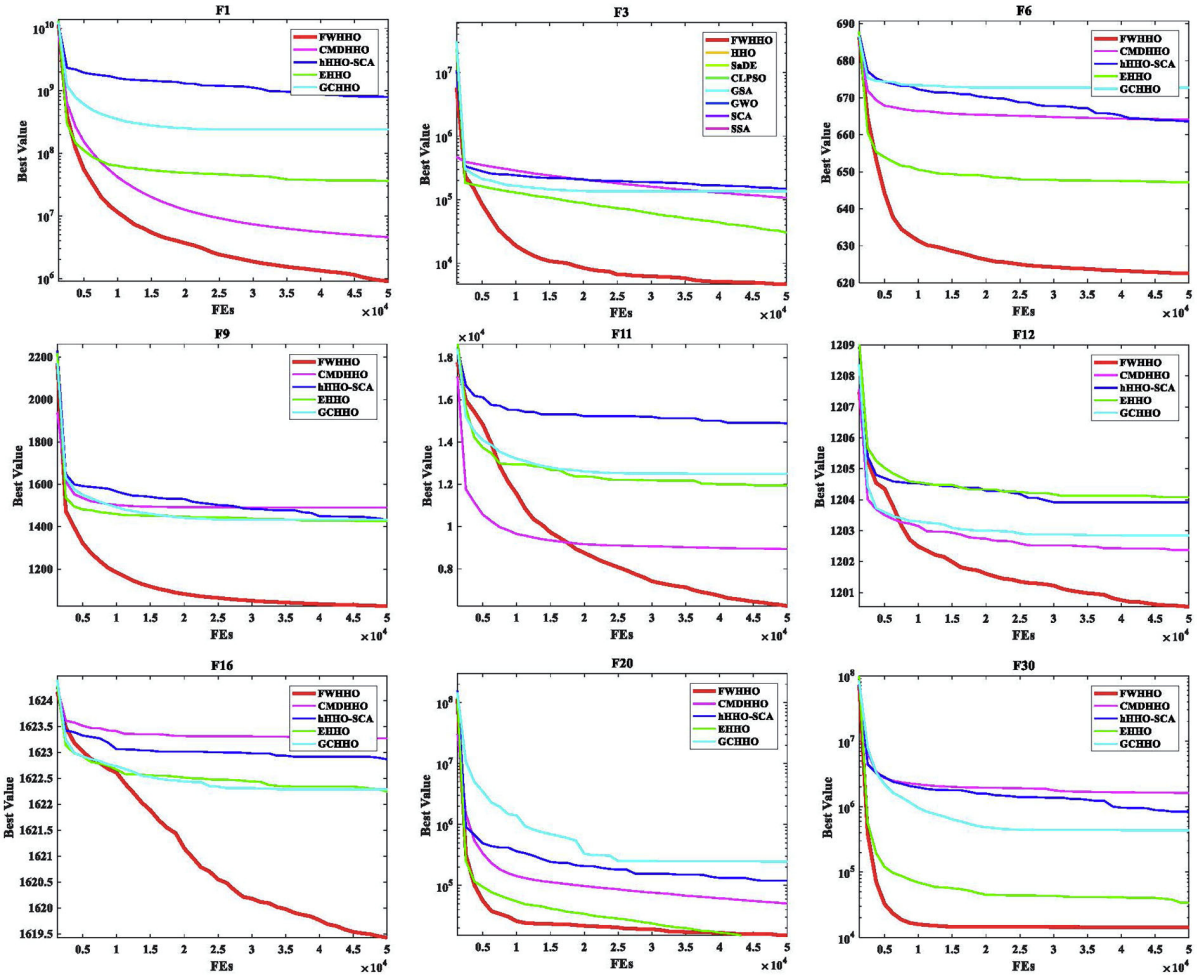
Convergence curves of FWHHO and existing FWA algorithms.

### 5.4. Application of FWHHO on machine learning evolution

The property of FWHHO with fireworks explosion has been verified based on the aforementioned experimental results. In this section, the FWHHO is used to evolve a kernel extreme learning machine (KELM) for the purpose of diagnosing COVID-19 using biochemical indexes. The classification performance of KELM depends entirely on two of the key parameters and the optimal feature subset (Shi et al., 2021). The FWHHO is used to optimize the parameters and subfeatures of KELM concurrently with biochemical indexes for diagnosing COVID-19. The data used in this experiment are the data we used and published earlier (Shi et al., 2021). A total of 51 patients with COVID-19 were included in the analysis retroactively between 21 January and 20 March 2020. Each patient with COVID-19 was evaluated for gender, age, biochemical index, and blood electrolyte values. Biochemical indices and blood electrolytes were determined using an automated biochemical analyzer at the clinical biochemistry laboratory at the Affiliated Yueqing Hospital of Wenzhou Medical University (BS-190; Mindray, Shenzhen, China). This data set consists of 25 biochemical index features. Moreover, several other state-of-the-art algorithms, such as ECPA, CPA, GWO, MFO, and PSO, are utilized to optimize the parameters and subfeatures of KELM; the original KELM, SVM, and KNN are used as comparisons, and 10-fold cross-validation is used in this work. Each algorithm is run independently 10 times diagnose COVID-19 utilizing biochemical indexes.

Comparison of the statistical results of the proposed FWHHO-KELM algorithm with existing competitive algorithms, such as ECPA-KEML, CPA-KELM, CPA-KELM, GWO-KELM, MFO-KELM, PSO-KELM, SVM, and KNN, is presented in Table 8. In comparison to existing algorithms, the suggested FWHHO-KELM has the best ACC (0.94320), MCC (0.92420), sensitivity (0.95354), specificity (0.90359), and sensitivity (0.95354). The best standard deviation is also obtained by the proposed FWHHO-KELM with 0.03854, 0.03584, 0.042511, and 0.05841. The results of the algorithm proposed in this article on this data set are higher than the results of the previous ECPA-KELM algorithm: 2.3%, 2.1%, 3.3%, and 0.8% in terms of four metrics. The original KELM and SVM algorithms perform poorly in the absence of swarm optimization algorithm evolution; however, the GWO-KELM, MFO-KELM, and PSO-KELM algorithms outperform the original KELM, SVM, and KNN algorithms. This experiment reveals that FWHHO-KELM can acquire the best property across all of these competing models automatically, owing mostly to the improved FWHHO, which can automatically select the optimal KELM parameters and subset of features for diagnosing COVID-19 using biochemical indices.

**Table 8:** The statistical study comparing outcomes in terms of the four criteria.

**Algorithms**	**ACC**	**MCC**	**Sensitivity**	**Specifity**
FWHHO-KELM	**0.94325 ± 0.03854**	**0.92426 ± 0.03584**	**0.95354 ± 0.042511**	**0.90359 ± 0.05841**
ECPA-KELM	0.92188 ± 0.04254	0.90507 ± 0.04145	0.92288 ± 0.05302	0.89637 ± 0.06523
CPA-KELM	0.87613 ± 0.06512	0.85305 ± 0.07865	0.86541 ± 0.06362	0.84225 ± 0.08965
KELM	0.80224 ± 0.07852	0.79652 ± 0.09863	0.80263 ± 0.06375	0.78557 ± 0.15452
GWO-KELM	0.86523 ± 0.06901	0.82512 ± 0.07524	0.87832 ± 0.07885	0.85263 ± 0.10152
MFO-KELM	0.85462 ± 0.06325	0.83661 ± 0.0705	0.88628 ± 0.08543	0.86360 ± 0.09016
PSO-KELM	0.85787 ± 0.06888	0.86327 ± 0.0757	0.87188 ± 0.06323	0.87858 ± 0.10325
SVM	0.81365 ± 0.08762	0.78631 ± 0.0888	0.78696 ± 0.08501	0.80266 ± 0.16214
KNN	0.81371 ± 0.08432	0.81374 ± 0.1136	0.78271 ± 0.08864	0.80188 ± 0.12426

Where bold indicates that the current value is the best in all algorithms.

In addition, the proposed FWHHO is utilized to optimize settings and choose optimal subfeatures for KELM concurrently to diagnose COVID-19 using biochemical indicators. In addition, the numbers of the selected features in each 10-fold cycle by these algorithms are shown in Table 9. As shown in Table 9, the FWHHO-KELM proposal clearly beats others, and in terms of statistics, the FWHHO-KELM picked the characteristics AGE, ALT, ALB, A/G, AST, and LDH with values of 9, 9, 9, 8, and 10, respectively, whereas the other features were chosen far less frequently. As a result of their frequent appearance, such qualities may aid in the early diagnosis of COVID-19 and the discrimination of other low-frequency features. Due to the underlying details in these frequency aspects, these AGE, ALT, ALB, A/G, AST, and LDH traits should be given additional care in clinical practice. In short, the proposed FWHHO can successfully crack numerical optimization problems.

**Table 9:** The numbers of selected feature.

**Index**	**Algorithm**					

	**FWHHO-KELM**	**ECPA-KELM**	**CPA-KELM**	**GWO-KELM**	**MFO-KELM**	**PSO-KELM**
F1	0	0	0	0	1	2
F2	9	9	7	8	8	7
F3	2	3	3	4	5	6
F4	3	4	5	3	3	5
F5	9	8	7	8	7	7
F6	1	2	4	5	5	4
F7	9	9	7	6	6	7
F8	0	2	3	4	5	4
F9	9	8	8	7	6	6
F10	4	5	4	4	6	4
F11	1	3	5	5	6	4
F12	8	9	8	7	7	8
F13	0	1	2	4	4	5
F14	10	8	7	7	6	6
F15	3	4	5	3	5	4
F16	5	6	5	2	4	3
F17	3	4	5	5	3	4
F18	1	2	5	5	3	6
F19	0	1	6	4	6	4
F20	2	4	5	4	5	3
F21	4	3	5	5	3	3
F22	0	2	4	6	4	4
F23	1	1	6	5	1	1
F24	0	2	4	4	2	6
F25	6	6	6	6	5	5

## 6. Conclusion and future work

The purpose of this research is to develop a novel HHO frame based on the fireworks explosions to enhance the performance of the original HHO for challenging numerical optimization tasks. The FWHHO framework suggests performing searches in two phases: first for hawks, then for fireworks explosions. Following the conclusion of the four stages of the hawks' search, a fireworks explosion search is performed to explore promising locations and potential food supplies. It then looks for adjacent fireworks explosions after selecting persons based on their proximity to one another. In addition, the dynamic amplitude is used to calculate the step size for searching for fireworks bursts. At first, the amplitude is considerable, allowing for exploration of prospective areas; as iteration progresses, the amplitude decreases, allowing for full exploitation of the space surrounding a potential solution. To be more precise, it selects several individuals based on their proximity to one another and then does a search for fireworks explosions in their vicinity. In addition, the dynamic amplitude is used to determine the step size of the search for fireworks explosions. At first, the amplitude is considerable, allowing for exploration of prospective locations; as iterations progress, the amplitude decreases, allowing for complete exploitation of the space surrounding a potential solution. Furthermore, FWHHO is compared with state-of-the-art algorithms, existing HHO algorithms, and existing fireworks algorithms, and the statistical findings demonstrate that FWHHO is superior in terms of solution quality and search efficiency.

There might be limitations to this research. The projected FWHHO may still have room for development. The proposed method, although effective, does need a significant amount of extra computing time and resources to implement. To this end, we will likely investigate ways to parallelize the implementation of the method in the near future. Complex engineering optimization issues, such as optimal control in industry and energy management, are prime candidates for the FWHHO algorithm.

## Data availability statement

The original contributions presented in the study are included in the article/supplementary material, further inquiries can be directed to the corresponding authors.

## Author contributions

MW: designing experiments, programming, and executing experiments. LC: revision, editing, software, visualization, and investigation. AH: algorithm design and experimental data statistics. HC: financial support, manuscript polishing, and provision of experimental equipment. All authors contributed to the article and approved the submitted version.
